# Comparison of single-pore non-liposuction near-infrared laparoscopy with conventional open surgery for axillary sentinel lymph node biopsy in patients with early breast cancer: a single-center, small-sample retrospective study

**DOI:** 10.1186/s12957-023-02942-w

**Published:** 2023-02-28

**Authors:** Cheng-cai Yao, Changchun Liu, Jiayi Xian

**Affiliations:** 1 Sixth Affiliated Hospital of South China University of Technology, and Sixth Clinical College of South China University of Technology, Foshan, 528225 People’s Republic of China; 2 Department of Breast Surgery, Sixth Affiliated Hospital of South China University of Technology, and Sixth Clinical College of South China University of Technology, Foshan, 528225 People’s Republic of China

**Keywords:** Early breast cancer, Indocyanide green, Carbon nanoparticle suspension, Sentinel lymph node biopsy, Near-infrared endoscopy

## Abstract

**Background:**

This study aimed to compare the effects of single-pore non-liposuction near-infrared (NIR) endoscopic surgery and traditional open surgery for axillary sentinel lymph node biopsy (SLNB) in patients with early breast cancer (EBC).

**Methods:**

The clinical pathological data of 61 patients with EBC who underwent axillary SLNB using indocyanine green (ICG) combined with carbon nanoparticle suspension (CNS) were retrospectively collected. Thirty patients received SLNB through single-pore non-liposuction NIR endoscopic surgery (endoscopic group), and the remaining 31 received SLNB through open-incision surgery (open group). The success rate, operation time, volume of intraoperative bleeding, postoperative axillary drainage, axillary extubation time, and the occurrence of postoperative complications were compared between the groups along with the total number of sentinel lymph nodes (SLNs), luminous SLNs, stained SLNs, and the pathological positivity rate of the SLNs.

**Results:**

All patients underwent SLNB with a 100% success rate. SLNB operation times of the endoscopic group were longer than those of the open group (*t* = 3.963, *P* = 0.000), and the volume of axillary drainage was inferior (*t* = 3.035, *P* = 0.004). However, there were no differences in the intraoperative bleeding volumes, axillary extubation times, and postoperative complications (*P* > 0.05). In the Open group, the mean number of SLNs was 5.12 ± 2.16, and the pathological positivity rate was 13.53%; in the Endoscopic group, these numbers were 4.89 ± 1.73 and 12.39%. The mean number of SLNs detected (*t* = 0.458, *P* = 0.649) and the pathological positivity rates (χ2 = 0.058, *P* = 0.810) did not differ between the two groups. All 61 patients were followed for a median of 14.6 months. There were no local recurrences or distant metastases.

**Conclusions:**

Our single-center results reveal that single-hole non-liposuction NIR endoscopic axillary SLNB is not inferior to open SLNB and may be an appropriate option for patients with early breast cancer who desire breast preservation with fewer incisions.

**Trial registration:**

This retrospective study was “retrospectively registered” at the Sixth Affiliated Hospital of South China University of Technology (no. 2020105) and in National Medical Research Registration and Archival Information System (https://www.medicalresearch.org.cn, number: MR-44-21-004727).

## Background

Endoscopic axillary lymph node surgery for early breast cancer (EBC) has historically been conducted using liposuction [1–5]. Liposuction allows smooth construction of the cavity, the axillary lymph nodes can be clearly displayed, and the difficulty of the surgery is reduced; thus, this technique is generally beneficial. However, liposuction increases axillary trauma, and its safety regarding the tumor is also questioned by some experts [6, 7]. Is it possible to perform an endoscopic sentinel lymph node biopsy (SLNB) using a non-liposuction method? We reviewed domestic and foreign literature and found several reports on this topic; however, most involved traditional endoscopic and single dyeing methods [8, 9], which is contrary to the fact that pigment combined with the fluorescence double dyeing method is widely used in domestic clinical practice to reduce the false negativity rate of SLNB [10]. In addition, the traditional endoscope has no near-infrared (NIR) fluorescence function, which limits its application as this form of fluorescence is used as a tracer in domestic mainstream SLNB. However, recently developed NIR endoscopes with fluorescence capabilities can address this issue. Can these be applied for non-liposuction SLNB? At present, no relevant reports have been published domestically or internationally, and only a few studies have been reported on SLNB by NIR endoscopy with the liposuction method [11]. The tumor safety of the liposuction method remains controversial [6, 7]. For this reason, we retrospectively analyzed the clinical data of early breast cancer patients with indocyanine green (ICG) fluorescence combined with a carbon nanoparticle suspension (CNS) as a tracer using a non-liposuction single-pore NIR endoscopic technique for axillary SLNB. The effects of this approach were compared with those of traditional open incision surgery in the same period to elucidate the efficacy and safety of this technique. This study was supported by the Medical Research Program of the Foshan Health and Family Planning Commission and the Foshan High-Level Medical Talent Training Fund, and approved by the ethics committee of the hospital (Approved on March 8, 2022, No.: 2022142).

### Data collection and processing

#### Source of cases

The clinical pathological data of 61 patients with EBC who underwent axillary SLNB using ICG combined with CNS from June 2020 to May 2022 were retrospectively collected. Thirty patients received SLNB through single-pore non-liposuction NIR endoscopic surgery (endoscopic group, EG), and additional 31 patients received SLNB through open incision surgery (open group, OG). The success rate, operation time, intraoperative bleeding volume, postoperative axillary drainage, axillary extubation time, and the occurrence of postoperative complications were compared between the groups. In addition, the number of the total sentinel lymph nodes (SLNs), luminous SLNs, stained SLNs, and the pathological positivity rate of these SLNs were assessed between the groups. The preoperative clinicopathology data for the two groups of patients are shown in Table 1.

**Table 1:** Preoperative clinicopathology datas for 2 groups of patients

	Open group	Endoscopic group	*t*/*χ*^2^ value	*P* value
Years (age, x ± s)	44.83 ± 2.34	45.12 ± 1.98	− 0.383	0.704
BMI (kg/m^2^)	25.18 ± 0.97	24.87 ± 1.25	0.018	0.986
Menstrual status (cases, %)				
Pre-menopause	16 (51.61)	14 (46.67)	0.180	0.981
Post-menopause	15 (48.39)	16 (53.33)		
Tumor size (cases, %)				
2 cm < T ≤ 3 cm	11 (35.48)	13 (43.33)	3.197	0.362
T ≤ 2 cm	20 (64.52)	17 (56.67)		
cTNM stages (cases, %)				
Istage	11 (35.48)	13 (43.33)	3.197	0.362
IIA stage	20 (64.52)	17 (56.67)		
ER expression (cases, %)				
Positive	18 (52.94)	19 (63.33)	2.934	0.402
Negative	13 (47.06)	11 (36.67)		
PRexpression (cases, %)				
Positive	14 (45.16)	12 (40.0)	1.492	0.684
Negative	17 (54.84)	18 (60.0)		
Her2expression(cases, %)				
Positive^a^	10 (32.26)	10 (33.33)	7.262	0.064
Negative^b^	21 (67.74)	20 (66.67)		
Ki67expression(例, %)				
≤ 14%	9 (29.03)	12 (40.02)	6.738	0.081
> 14%	22 (70.97)	18 (60.0)		
Molecular type				
Luminal type	16 (51.61)	15 (50.0)	10.902	0.053
Her2 positive type	10 (32.26)	10 (33.33)		
TNBC type	5 (16.13)	5 (16.67)		

^a^Positive: IHC test 3+, 2+ but Fish test positive

^b^Negative: IHC test negative, 1+ and 2+ but Fish test negative

### Inclusion criteria

1. Patients with invasive breast ductal carcinoma confirmed by preoperative pathology;
2. Patients without neoadjuvant therapy and cT ≤ 3.0 cm N0M0;
3. Patients received breast-conserving surgery, and axillary SLNB was performed with ICG fluorescence combined with CNS double staining method.

### Criteria for exclusion

1. Patients with inflammatory breast cancer and carcinoma *in situ*;
2. Patients with cT > 3.0 cmN_0-3_M_0-1_;
3. Patients who had undergone neoadjuvant therapy;
4. Patients with a medical history of ipsilateral axillary surgery, radiotherapy, or lymphatic disease;
5. Modified radical mastectomy, simple mastectomy, and axillary SLNB were performed by a single-tracer method or combined radionuclide staining method;
6. Patients allergic to ICG and CNS;
7. Endoscopic axillary SLNB was performed by the liposuction method.

### Reagents and instruments

Indocyanine green for injection, specification: 25 mg, manufactured by Dandong Zhichuang Pharmaceutical Co., Liaoning Province, China. Carbon nanoparticle suspension, specifications: 0.5 ml: 25 mg, manufactured by Chongqing Laimei Pharmaceutical Co., Ltd., Chongqing City, China. The fluorescence vein imager was provided by Harbin Haihongji Technology Development Co. The 4k ultra-HD laparoscopic NIR imaging system (DPM-ENDOCAM-03 and DPM-LIGHT-03, Zhuhai, China) was from Dipu Medical Technology Co.

### The methods of the SLNB

**Fig. 1:**
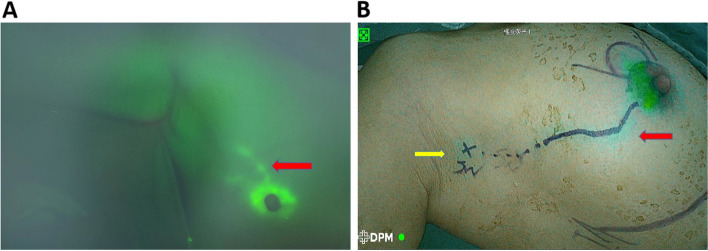
Fluorescent subcutaneous lymph vessels (red arrows) and the marked SLN at the body surface (yellow arrow points). **A** Fluorescence vascular image and **B** 4k HD NIR laparoscopy

The patient was sedated, and 10–15 min prior to surgery, 0.5 mL of 0.5 mg/mL diluted ICG solution was administered subcutaneously at the 12 o’clock and 6 o’clock positions in the areola of the affected side, and the injection site was gently rubbed for 5 min. Then, 0.5 mL of 50% CNS was injected subcutaneously at the 3 o’clock and 9 o’clock positions in the areola of the affected side, and the site was gently rubbed for 5 min [11]. The subcutaneous ICG luminous-lymphatic vessels (Fig. 1A) were imaged using a fluorescent vascular imager (China Harbin Haihong Technology Development Co. LTD.) or 4K HD NIR fluorescent endoscope (China Dipu Medical Technology Co.), and the body surface was marked with a pen (Fig. 1B). The surface projection of the SLN was marked 1.0–1.5 cm distal to the ICG luminous-lymphatic vessel.

#### Open incision axillary SLNB

**Fig. 2:**
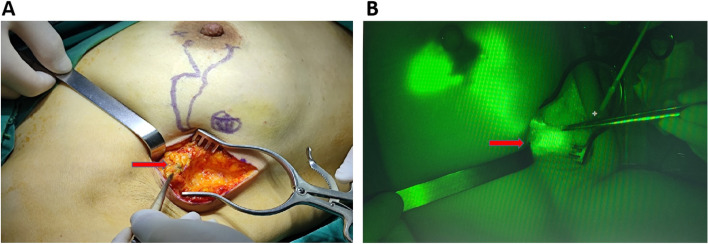
SLNs are shown during the SLNB with open incisions. **A** CNS-stained SLNs and **B** ICG fluorescent SLNs (red arrow)

A 4–5 cm incision was made in the affected axilla at the surface mark of the SLN, the skin and subcutaneous adipose tissue were cut, and the fascia of the axilla was opened. Under the real-time guidance of the fluorescence vessel imager, stained SLNs (Fig. 2A) or/and fluorescent SLNs (Fig. 2B) were traced along the stained or luminous lymphatic vessels. All fluorescent and stained lymph nodes were removed during the procedure and sent for examination as SLNs. The axilla was then probed with the finger to locate enlarged lymph nodes, which were also removed as non-sentinel lymph nodes (NSLNs). Axillary lymph node dissection (ALND) was routinely performed if the SLNs were positive. Subsequently, a 5–6-cm excision was made on the surface of the breast tumor, and the tumor tissue and surrounding normal glandular tissue were completely removed with an approximately 1.0 cm margin to complete the breast preservation procedure for early breast cancer.

#### NIR endoscopic axillary SLNB

**Fig. 3:**
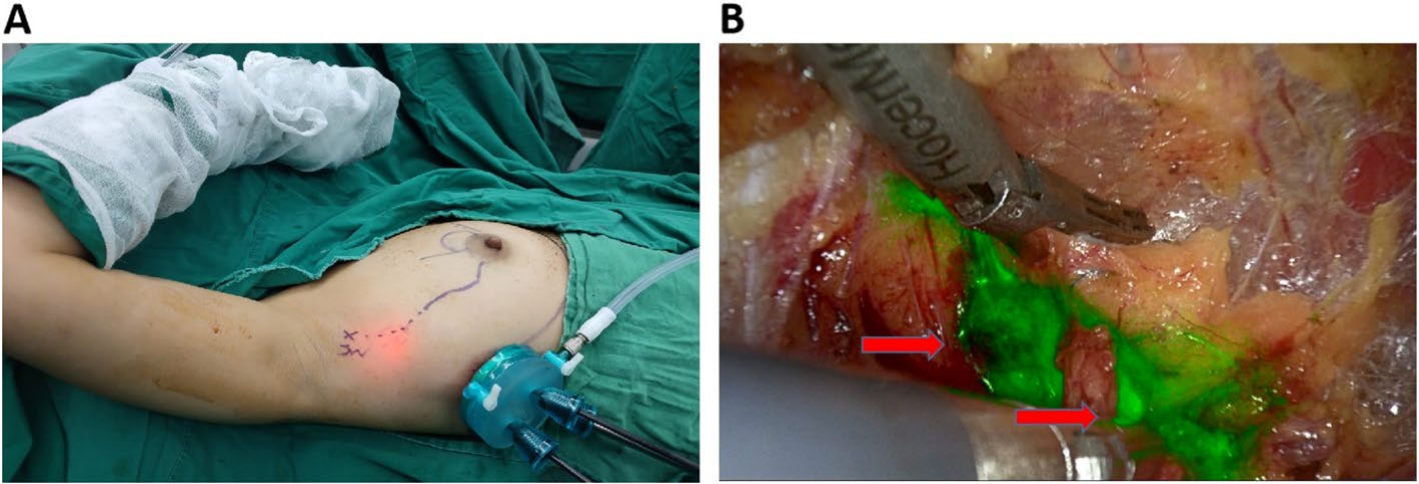
Axillary SLNB by NIR endoscopy. **A** Surgical procedure performed by a single insertion of a single-pore endoscopic retractor fixator through the thoracic transaxillary midline. **B** Green fluorescent SLNs (red arrow) by NIR endoscopy

A single 3–4 cm incision was made across the fourth to sixth intercostal spaces at the midaxillary line, and the skin and subcutaneous adipose tissue were cut open and sharply separated to the armpit area. When the cavity was sufficiently opened, a disposable incision protection retractor fixator was placed, filling the cavity with CO_2_ gas at a flow rate of 20 L/min and pressure maintained at 8–10 mmHg to provide the space required for the surgery (Fig. 3A). Next, the endoscope was adjusted to near-infrared mode, and sharp separation was performed along the superficial fascia to the surface projection of the axillary SLN (labeled before surgery) without liposuction. The intercostal nerve was first seen in the dissociation process and retained during the operation. Dissection was then performed along the upper side to the lateral border of the pectoralis major in the axillary region. Fluorescent or pigmented lymphatic vessels were often seen at this time and were followed to locate the axillary SLNs. All fluorescent and stained lymph nodes were resected as SLNs, and the surrounding non-fluorescent and non-stained lymph nodes were resected as NSLNs (Fig. 3B). If the SLNs were positive, ALND was performed by endoscopic surgery. Then, through the same incision, the tumor and approximately 1.0 cm of surrounding normal glandular tissue were completely removed, completing the breast preservation procedure for early breast cancer.

### Judgment of pathologically positive lymph nodes

According to the professional guidelines and regulations of breast cancer of the Chinese Anti-Cancer Association (2017 edition) [12], the SLNs, NSLNs, and the dissected axillary lymph nodes were examined by pathological paraffin sections during the operation, and those with macrometastasis, micrometastasis, and isolated tumor cells were considered positive.

### Statistical methods

Data were analyzed using the software package SPSS, version 21.0. A *t* test was used to compare measurement data, and a *χ*2 test or Fisher’s exact test was used to compare count data. *P* < 0.05 was considered to indicate significance. The number of detected SLNs, detection rates, and positive pathology rates in both groups were evaluated using postoperative routine paraffin pathology protocols.

## Results

### Surgical status and postoperative complications in the two groups

The axillary SLNB procedure was completed in both groups with a 100% success rate. Compared with the Open SLNB group, operation times of the Endoscopic group were longer (*t* = 3.963, *P* = 0.000) and the volume of axillary drainage was lower (*t* = 3.035, *P* = 0.004), and the differences were statistically significant. However, there were no differences in intraoperative blood loss, axillary extubation time, and postoperative complications (poor incision healing, edema of the affected upper limb, and limited functional activity of the affected upper limb) between the two groups. Refer to Table 2 for details.

**Table 2:** Surgical and post-operative complications in 2 groups of patients

	Open group	Endoscopic group	*t*/*χ*^2^ value	*P* value
Operation times (min)	30.29 ± 6.15	35.36 ± 3.41	3.963	0.000
Volume of intraoperative bleeding (ml)	5.63 ± 3.78	5.46 ± 2.31	0.211	0.834
Volume of axillary total drainage (ml)	255.62 ± 8.28	248.53 ± 9.92	3.035	0.004
Axillary extubation times (days)	5.16 ± 1.95	4.98 ± 1.72	0.382	0.704
Postoperative complications (*n*/%)				
Poorly incision healing	2 (6.45)	1 (3.33)	0.317^a^	0.573^a^
Limited of the upper extremity function	0 (0.00)	0 (0.00)	–	1.000^b^
Edema of the upper extremities	0 (0.00)	1 (3.33)	–	0.492^b^

^a^The Yates’ continuous correction *χ*2 test

^b^The Fisher exact probability method

### Detection of axillary SLNs and pathologically positive SLNs in both groups

A total of 133 SLNs were detected in the Open group, with a mean of (5.12 ± 2.16). Among them, 18 SLNS were pathologically positive, with a positivity rate of 13.53% (18/133). A total of 121 SLNs were detected in the Endoscopic group, with a mean of (4.89 ± 1.73), and 15 SLNs were pathologically positive, with a positivity rate of 12.39% (15/121). The total number of SLNs detected (*t* = 0.458, *P* = 0.649) and the pathological positivity rate of the detected SLNs (*χ*^2^ = 0.058, *P* = 0.810) did not differ between the two groups. In the open group, 112 fluorescent SLNs (86 both fluorescent and stained SLNs + 26 pure fluorescent SLNs) and 97 stained SLNs (86 both fluorescent and stained SLNs + 11 pure stained SLNs) were detected, and there was no significant difference between these SLNs (*χ*^2^ = 3.778, *P* = 0.052). In the Endoscopy group, 106 fluorescent SLNs (73 both fluorescent and stained SLNs + 33 pure fluorescent SLNs) and 88 stained SLNs (73 both fluorescent and stained SLNs + 15 pure stained SLNs) were detected, and the difference was statistically significant (*χ*^2^ = 6.953, *P* = 0.008). In the Open group, 17 of the 18 pathologically positive SLNs were fluorescent and 16 were stained, and there was no significant difference between them (*χ*^2^ = 0.364, *P* = 0.547). In the endoscopy group, all 15 pathologically positive SLNs were fluorescent and 13 were stained, and there was no significant difference between them (*P* = 0.483). Additional analyses showed no differences in the detection rates of pathologically positive fluorescent SLNs (*P* = 0.483) and pathologically positive stained SLNs (*χ*2 = 0.038, *P* = 0.846) between the two groups, as shown in Table 3.

**Table 3:** Detection rate of luminescence-SLNs, staining-SLNs, and pathological positive rates in the 2 groups (%, n/N)

	Open group	Endoscopic group	χ^2^ value	*P* value
Luminescence-SLNs	84.21 (112/133)	87.60 (106/121)	0.475	0.491
Staining-SLNs	72.93 (97/133)	72.73 (88/121)	0.001	0.975
*χ*^2^ value	3.778	6.953		
*P* value	0.052	0.008		
Pathological positive luminescence-SLNs	94.44 (17/18)	100 (15/15)	–	1.000^b^
Pathological positive staining-SLNs	88.89 (16/18)	86.67 (13/15)	0.038^a^	0.846^a^
*χ*^2^ value	0.364^a^	–		
*P* value	0.547^a^	0.483^b^		

*Luminescence-SLNs* both luminescence and staining-SLNs + pure luminescence-SLNs; *staining-SLNs* both luminescence and staining-SLNs + pure staining-SLNs

^a^The Yates’ continuous correction *χ*2 test

^b^The Fisher exact probability method

### Postoperative management

After the procedure, the breast and axillary surgical areas were bandaged locally with pressure, and the volume of axillary drainage was measured. The drain was removed when the volume of axillary drainage fell below 15–20 mL/day for three consecutive days. Depending on the pathology results, chemotherapy, targeted therapy, and endocrine therapy were performed after the surgery. A total of 61 patients received radiotherapy within 8 weeks of surgery.

### Management of postoperative complications

Two patients with poor wound healing in the open group and one in the endoscopic group were cured with surgical dressing changes within 1 month. One patient in the endoscopic group had postoperative upper limb function restriction and a score of 56 on the Disabilities of the Arm, Shoulder, and Hand questionnaire (DASH) [13]. The patient recovered within 2 months with acupuncture and rehabilitation exercises.

### Follow-up status

Sixty-one patients were followed up for 1–24 months, and none were lost to follow-up (follow-up rate of 100%). There were no local recurrences or distant metastases during the mean follow-up of 14.6 months.

## Discussion

Endoscopic technology is widely used in gynecology [14], gastrointestinal surgery [15], hepatobiliary surgery [16], cardiothoracic surgery [17, 18], and thyroid surgery [19]; however, it is relatively rare in breast surgery and is mostly limited to gynecomastia [20] and axillary lymph node dissection [1–5]. The reason may be the lack of natural cavities in the breast and axilla. The foundation for the successful implementation of endoscopic surgery is the establishment and maintenance of an operating space, which is a challenge in breast surgery. However, in recent years, through exploration by domestic and foreign scholars, endoscopic technology for breast surgery [21, 22] has been greatly developed, and some hospitals have even conducted robotic breast surgery [23, 24].

In this study, 30 patients with early-stage breast cancer in the Endoscopy group were treated with ICG fluorescence in combination with CNS pigment double-dyeing and received axillary SLNB from the axillary midline approach of the transverse thorax using a single-hole non-liposuction method. The SLNB operation had a 100% success rate. The mean operation time, the volume of intraoperative bleeding, the postoperative axillary extubation time, and the complications (poor wound healing, upper limb function restriction, and upper limb edema) did not differ between the open and endoscopic groups. Notably, the endoscopic surgery time was not significantly longer than the open surgery time. The results revealed that the volume of axillary drainage after surgery was greater in the open group than in the Endoscopic group, and this difference was significant. This finding may be due to the ultrasonic knives used for dissection during endoscopic surgery, which are more favorable than the high-frequency electrospray knives used to close the tiny lymphatic vessels in the axilla, reducing the probability of lymphatic leakage. However, one patient in the Endoscopic group had mild to moderate impairment of upper arm function on the affected side after surgery, with a DASH score of 56. The reason may be that endoscopic axillary SLNB surgery is not mature enough at an early stage, the operation time is relatively long, and the upper arm of the affected side is excessively tightly bandaged. The “full moon” position, which retains elbow flexion and elevation on a head frame for a prolonged time, is related to the patient’s forearm musculocutaneous nerve injury, which recovered within 2 months after surgery after acupuncture and intermediate frequency pulse electrical stimulation treatments. These findings suggest that single-hole non-liposuction NIR endoscopy for SLNB in early-stage breast cancer patients is as safe and feasible as open surgery, with manageable complications.

At present, endoscopic techniques for SLNB in early breast cancer are mostly performed in the context of axillary liposuction [1–5]. The anatomy of the axilla becomes clearer with liposuction, and stained lymph nodes, fluorescent lymph nodes, and radionuclide-labeled lymph nodes can be more visually displayed in the axilla, thus reducing the difficulty of SLNB and increasing its accuracy rate. However, liposuction increases trauma due to deep depression of the axilla, which affects the patient’s appearance, which some patients find difficult to accept. In addition, whether liposuction can cause tumor cell dissemination, especially for patients with potential axillary lymph node positives, is controversial [6, 7].

To avoid such controversy, this study explored the use of non-liposuction techniques for axillary SLNB. It is difficult to construct axillary cavities and accurately locate SLNs with minimal trauma without liposuction. To address this issue, we used high-frequency color ultrasound to locate and label axillary SLNs prior to surgery and a fluorescence vascular imager combined with a 4K high-definition near-infrared endoscopic method to further locate SLNs during surgery, with excellent results. The 4K HD NIR endoscope has both the white light mode of a conventional endoscope and the fluorescence mode of the endoscope itself. Therefore, the detection rate of SLNs can be improved by tracing pigments in combination with fluorescence double staining to detect both stained and fluorescent lymph nodes during SLNB operation.

In this study, we successfully detected SLNs within approximately 30 min in 30 patients in the Endoscopy group using the NIR endoscopic fluorescence method to accurately locate SLNs prior to surgery; this surgery was performed with a 100% success rate. A total of 121 SLNs were detected in the Endoscopic group (mean 4.89 ± 1.73), significantly exceeding the “no less than 3” requirement of the guidelines for reducing the false negativity rate [25, 26]. These findings were equivalent to those in the open group. The detection rates of fluorescent SLNs and stained SLNs did not differ between the endoscopic and open groups. However, it is interesting to note that there was a difference between the detection rates of fluorescent SLNs and stained SLNs within the endoscopic group. The reasons for investigating this are that the molecular diameter of the ICG is smaller than that of the CNS, which allows it to quickly penetrate smaller lymphatic vessels and expand to further lymph nodes. In addition, this finding indicates that 4K high-definition NIR endoscopy has superior fluorescence sensitivity, so much so that additional faint fluorescent lymph nodes can be found. Although the number of fluorescent SLNs was greater than the number of stained SLNs in the Endoscopy group, there was no difference in the pathological positivity rates between fluorescent SLNs and stained SLNs, indicating that it is safe to use either ICG fluorescence or CNS pigments as tracers in axillary SLNB.

Sixty-one patients were followed up for 1–24 months, and none were lost to follow-up. No local recurrences or distant metastases occurred during a mean follow-up period of 14.6 months. Similar to the findings of previous studies [27, 28], the oncology safety and reliability of the non-liposuction fluorescent endoscopic technique for axillary SLNB in early breast cancer was demonstrated.

This was a single-center, single-arm study; thus, the sample size was not large, and the follow-up time was not long. Additional shortcomings may also have affected the study results. We plan to continue to expand the sample size, reduce the bias, and obtain additional positive results.

## Conclusions

In conclusion, based on our single-center results, single-hole non-liposuction NIR endoscopic axillary SLNB is not inferior to open SLNB and may be an appropriate option for patients with early-stage breast cancer who wish to undergo breast preservation with fewer incisions. It is recommended that this study be conducted at different centers, including more subjects.

## Data Availability

The datasets used or analyzed during the current study are available from the corresponding author upon reasonable request.

## Abbreviations

NIR: Near-infrared
SLNB: Sentinel lymph node biopsy
EBC: Early breast cancer
ICG: Indocyanine green
CNS: Carbon nanoparticle suspension
SLNs: Sentinel lymph nodes
NSLNs: Non-sentinel lymph nodes
ALND: Axillary lymph node dissection
DASH: The scores of Disabilities of the Arm, Shoulder, and Hand questionnaire
